# Alcohol consumption during pregnancy and birth outcomes: the Kyushu Okinawa Maternal and Child Health Study

**DOI:** 10.1186/1471-2393-14-79

**Published:** 2014-02-20

**Authors:** Yoshihiro Miyake, Keiko Tanaka, Hitomi Okubo, Satoshi Sasaki, Masashi Arakawa

**Affiliations:** 1 Department of Preventive Medicine and Public Health, Faculty of Medicine, Fukuoka University, Fukuoka, Japan; 2 Department of Social and Preventive Epidemiology, Graduate School of Medicine, The University of Tokyo, Tokyo, Japan; 3 Health Tourism Research Center, Graduate School of Tourism Sciences, University of the Ryukyus, Okinawa, Japan

## Abstract

**Background:**

A recent meta-analysis showed no relationships between light to moderate alcohol consumption during pregnancy and the risk of low birth weight (LBW), preterm birth (PTB), or small-for-gestational-age (SGA). Here, we present the first epidemiological study on this topic in Japan.

**Methods:**

Study subjects were 1565 Japanese mothers with singleton pregnancies and the babies born from these pregnancies. Alcohol consumption during pregnancy was assessed using a self-administered diet history questionnaire. Alcohol consumption during pregnancy was classified into three categories (none, < 1 g/day, and ≥ 1 g/day).

**Results:**

The mean birth weight of the babies was 3006.3 g. 7.7% were classified as LBW, 4.0% as PTB, and 7.8% as SGA. The range of maternal alcohol consumption during pregnancy was 0.0 to 11.7 g per day: 1356 (86.7%) mothers were abstainers and the 95th percentile value was 0.84 g per day. Compared with abstinence, alcohol consumption of 1.0 g or more per day during pregnancy was significantly associated with an increased risk of PTB with a significant positive linear trend: the adjusted OR for PTB associated with maternal alcohol consumption of 1.0 g or more per day was 2.58 (95% CI: 1.004 - 5.80, *P* for trend = 0.03). No significant relationships were observed between maternal alcohol consumption during pregnancy and the risk of LBW or SGA, and there was no material association between maternal alcohol consumption during pregnancy and birth weight.

**Conclusions:**

This is the first study in Japan to show that maternal alcohol consumption during pregnancy of 1.0 g or more per day was significantly positively associated with the risk of PTB, but not LBW or SGA.

## Background

A systematic review by Henderson et al. found no convincing evidence that maternal alcohol consumption during pregnancy at low to moderate levels has adverse effects on miscarriage, stillbirth, intrauterine growth restriction, prematurity, birth weight, small-for-gestational-age (SGA), and birth defects including fetal alcohol syndrome [1]. A recent meta-analysis by Patra et al. revealed that heavy maternal alcohol consumption during pregnancy increases the risk of low birth weight (LBW), preterm birth (PTB), and SGA, whereas light to moderate alcohol consumption shows no effect: compared with abstainers, the exposure-response associations of maternal alcohol consumption during pregnancy with the risk of LBW and SGA were not apparent up to 10 g pure alcohol per day, and maternal alcohol consumption during pregnancy of less than 19 g pure alcohol per day was not associated with the risk of PTB; at higher consumption levels, risk of LBW, SGA, and PTB increased linearly with maternal consumption. It should be noted, however, that no studies conducted in Asian countries were included in this meta-analysis [2]. Four studies in the USA have shown significant positive relationships between low to moderate maternal alcohol consumption during pregnancy and the risk of LBW, PTB, and/or SGA [3-6], while equally significant inverse associations have been observed in two studies in Canada and Spain [7,8]. To our knowledge, there have been only three previous studies on alcohol consumption during pregnancy and birth weight or LBW in Japan, none of which have revealed any associations [9-11]; however, no epidemiological studies in Japan have examined the association of maternal alcohol consumption during pregnancy with the risk of PTB and SGA. Therefore, we wished to investigate this issue using data from the Kyushu Okinawa Maternal and Child Health Study (KOMCHS).

## Methods

### Study population

The KOMCHS is a prospective prebirth cohort study. Details of the baseline survey of the KOMCHS have been described elsewhere [12]. Eligible women were those who became pregnant while living in one of seven prefectures on Kyushu Island in southern Japan, with a total population of approximately 13.26 million, or in Okinawa Prefecture, an island chain in the southwest of Japan, with a total population of nearly 1.37 million. Between April 2007 and March 2008, we asked 423 obstetric hospitals in the abovementioned eight prefectures to provide a set of leaflets explaining the KOMCHS, an application form to participate in the study, and a self-addressed and stamped return envelope directly to as many pregnant women as possible. Pregnant women who were willing to participate in the study returned the application form to the data management center. A total of 1757 pregnant women between the 5th and 39th weeks of pregnancy gave their written informed consent to participate in the KOMCHS and completed the baseline survey. Of the 1757 women, 1590 mother-child pairs took part in the second survey, which was carried out after delivery: 73.5% of the mothers completed the second survey less than one month after giving birth, while 12.9%, 7.4%, 3.3%, and 2.9% completed the second survey at one, two, three, and 4–11 months after giving birth, respectively. Excluded from the current analysis were 23 mothers with multiple births and two mothers for whom the gender of the baby was not known due to incomplete data reporting. The final analysis included 1565 mother-child pairs. The ethics committee of the Faculty of Medicine, Fukuoka University approved the KOMCHS.

### Measurements

In each survey, a self-administered questionnaire was used. Participants filled out these questionnaires and mailed them to the data management center at the time of each survey. Research technicians completed missing or illogical data by telephone interview.

The first part of the first questionnaire at baseline elicited information about maternal age; region of residence; number of children; family structure; maternal education; and maternal employment status. Women were classified as unemployed if they were unemployed in both the year in which the questionnaire was completed and the preceding year. The second part of the first questionnaire was a semi-quantitative, comprehensive dietary history questionnaire (DHQ) that assessed dietary habits during the month preceding the completion of the questionnaire [13,14]. Estimates of daily intake of foods (total of 150 foods), energy, and selected nutrients were calculated using an ad hoc computer algorithm for the DHQ based on the Standard Tables of Food Composition in Japan [15]. The DHQ included questions on consumption of six types of alcoholic beverages: beer, Japanese sake (rice wine), shochu (a distilled alcoholic beverage made in Japan), chuhai (made with shochu and carbonated water), whisky, and wine. Consumption of each type was calculated based on the reported consumption frequency per week or per month and 12 portion sizes. Estimated average amounts of alcohol consumed per day for all beverage types were summed, yielding an average daily total alcohol intake in g/day. In a validation study of 92 Japanese women, the Spearman’s correlation coefficient between the DHQ and 16-day weighed dietary records was 0.74 for alcohol (S. Sasaki, unpublished observations, 2006). Body weight and height were self-reported as part of the DHQ. Body mass index was calculated as weight (kg) divided by the square of height (m).

In Japan, generally, an obstetrician’s estimate of gestational age at the time of delivery is based on early ultrasound examination and/or the first day of the last menstrual period, and birth weight is measured immediately after birth. Data such as birth weight and gestational age at birth are recorded by staff at the birth hospital or clinic in a booklet called the Maternal and Child Health Handbook. A copy of this booklet is provided to every pregnant woman by the municipality in which she resides at the start of her pregnancy; it is used to record data pertaining to prenatal checkups, postnatal health condition of both mother and baby, and growth of the child.

The second self-administered questionnaire elicited information on active maternal smoking status in the first (≤ 15 weeks’ gestation), second (16–27 weeks’ gestation), and third (≥ 28 weeks’ gestation) trimesters; gestational age at birth; birth weight; and baby’s gender. With regard to gestational age at birth and birth weight, mothers were advised to refer to their Maternal and Child Health Handbook before filling in our questionnaire.

LBW was defined as a birth weight less than 2500 g. PTB was defined as a birth occurring at a gestational age of less than 37 weeks. SGA was defined as a birth weight below the 10th percentile of the Japanese neonatal anthropometric norms for babies of the same gestational age, gender, and parity published by Itabashi et al. in 2010 [16]. These norms show the distribution of birth weights for each day of gestational age; in this study, however, data on gestational ages were only available in weeks. Thus, for the purposes of comparison and analysis, we selected the distributions from the third day of each week from the study of Itabashi et al.

### Statistical analysis

Maternal age at the time of the first questionnaire; region of residence; number of children; family structure; maternal education; maternal employment; body mass index; maternal smoking during pregnancy; gestational age at birth; and baby’s gender were selected as a priori potential confounding factors; however, gestational age at birth was removed when associations with the risk of LBW and PTB were examined. Marital status was not taken into consideration because 96.3% of the mothers lived with their husbands and there was only one single mother in the study. Alcohol consumption during pregnancy was classified into three categories (none, < 1 g/day, and ≥ 1 g/day); region of residence into three (Fukuoka Prefecture, prefectures other than Fukuoka Prefecture on Kyushu Island, and Okinawa Prefecture); number of children previously born to the same mother into three (0, 1, and 2 or more); family structure into two (nuclear and extended); maternal education into three (< 13 years, 13–14 years, and ≥ 15 years); maternal employment into two (yes and no); and maternal smoking during pregnancy into four (never smoked during pregnancy, smoked only in the first trimester, smoked in the second and/or third trimesters regardless of smoking status in the first trimester but not throughout the pregnancy, and smoked throughout pregnancy). Maternal age, body mass index, and gestational age at birth were used as continuous variables.

Adjusted odds ratios (ORs) and 95% confidence intervals (CIs) for birth outcomes in relation to maternal alcohol consumption during pregnancy were calculated through multiple logistic regression analysis. Analysis of covariance was used to calculate adjusted means of birth weights according to maternal alcohol consumption during pregnancy with allowance for confounding factors. Trends of an association were assessed with a multiple logistic regression model or a multiple linear regression analysis assigning consecutive integers to the categories of alcohol consumption. The interaction was tested using a term of the product of two variables in a multiple logistic regression model. All computations were performed using the SAS software package version 9.2 (SAS Institute, Inc., Cary, NC, USA).

## Results

Among the 1565 mother-child pairs, maternal mean age at baseline was 31.3 years (Table 1). According to the second questionnaire, 2.5% of mothers had smoked throughout their pregnancy and 91.2% had never smoked during pregnancy. The mean birth weight of the babies was 3006.3 g. 7.7% were classified as LBW, 4.0% as PTB, and 7.8% as SGA. The range of maternal alcohol consumption during pregnancy was 0.0 to 11.7 g per day: 1356 (86.7%) mothers were abstainers and the 95th percentile value was 0.84 g per day; therefore, there were no heavy drinkers (defined as more than 14 g per day).

**Table 1:** Distribution of selected characteristics in 1565 mother-child pairs

**Variable**	***n *** **(%)**
Baseline characteristics	
Maternal age, years, mean ± SD	31.3 ± 4.2
Region of residence	
Fukuoka Prefecture	883 (56.4)
Prefecture on Kyushu Island other than Fukuoka	527 (33.7)
Okinawa Prefecture	155 (9.9)
Number of living children already born to same mother	
0	615 (39.3)
1	633 (40.5)
≥ 2	317 (20.3)
Nuclear family structure	1336 (85.4)
Maternal education, years	
< 13	361 (23.1)
13 - 14	523 (33.4)
≥ 15	681 (43.5)
Maternal employment^a^	943 (60.3)
Body mass index, kg/m^2^, mean ± SD	21.4 ± 2.7
Characteristics in the second survey	
Maternal smoking during pregnancy	
None	1427 (91.2)
First trimester only	71 (4.5)
Second and/or third trimesters but not throughout	28 (1.8)
Throughout	39 (2.5)
Birth weight, g, mean ± SD	3006.3 ± 395.5
Gestational age, weeks, mean ± SD	38.9 ± 1.5
Male gender	762 (48.7)
Low birth weight (< 2500 g)	120 (7.7)
Preterm birth (< 37 weeks)	62 (4.0)
Small-for-gestational-age (< 10th percentile)	122 (7.8)

^a^Full-time or part-time employment in the year when the first questionnaire was conducted or in the previous year.

Table 2 shows the distributions of confounding factors according to maternal alcohol consumption during pregnancy. Maternal alcohol consumption during pregnancy was positively associated with living in Fukuoka Prefecture, number of children, body mass index, and maternal smoking throughout pregnancy and inversely with maternal education and gestational age.

**Table 2:** Characteristics of 1565 mother-child pairs categorized according to alcohol consumption during pregnancy

	**Alcohol consumption during pregnancy**			
**Variable**	**None (n = 1356)**	**< 1.0 g/day (n = 137)**	**≥ 1.0 g/day (n = 72)**	***P *** **for trend**^**a**^
Age, years, mean	31.2	31.4	31.6	0.48
Region of residence, %				0.78
Fukuoka Prefecture	56.3	54.7	61.1	
Other than Fukuoka Prefecture in Kyushu	33.5	38.0	29.2	
Okinawa Prefecture	10.2	7.3	9.7	
Number of children, %				0.01
0	39.8	36.5	34.7	
1	41.2	38.0	31.9	
≥ 2	19.0	25.6	33.3	
Nuclear family structure, %	85.6	81.8	87.5	0.78
Maternal education, years, %				0.01
< 13	22.4	21.2	38.9	
13 - 14	33.6	31.4	33.3	
≥ 15	44.0	47.5	27.8	
Maternal employment^b^, %	59.8	62.0	65.3	0.31
Body mass index, kg/m^2^, mean	21.3	21.8	21.9	0.02
Maternal smoking during pregnancy, %				< 0.0001
None	92.3	89.1	73.6	
First trimester only	4.1	5.8	11.1	
Second and/or third trimesters but not throughout	1.6	1.5	5.6	
Throughout	2.0	3.7	9.7	
Gestational age, weeks, mean	38.9	38.5	38.8	0.04
Male gender, %	49.0	43.1	52.8	0.85

^a^For continuous variables, a linear trend test was used; for categorical variables, a Mantel-Haenszel χ^2^-test was used.

^b^Full-time or part-time employment in the year when the first questionnaire was conducted or in the previous year.

Table 3 gives adjusted ORs and the 95% CIs for LBW, PTB, and SGA and adjusted mean birth weights in relation to maternal alcohol consumption during pregnancy. Compared with abstainers during pregnancy, pregnant women who had consumed 1.0 g or more of alcohol per day during pregnancy had a significantly increased risk of PTB, and the exposure-response association between maternal alcohol consumption during pregnancy and the risk of PTB was significant, although a positive relationship between maternal alcohol consumption of less than 1.0 g per day during pregnancy and PTB was not statistically significant: the adjusted OR for PTB associated with maternal alcohol consumption of 1.0 g or more per day was 2.58 (95% CI: 1.004 - 5.80, *P* for trend = 0.03). No significant relationships were observed between maternal alcohol consumption during pregnancy and the risk of LBW or SGA. Also, there was no material association between maternal alcohol consumption during pregnancy and birth weight.

**Table 3:** Adjusted odds ratios (ORs) and 95% confidence intervals (CIs) for birth outcomes and adjusted means of birth weight in relation to alcohol consumption during pregnancy in 1565 mother-child pairs

**Alcohol consumption during pregnancy**	**Low birth weight**		**Preterm birth**		**Small-for-gestational-age**		**Adjusted mean of birth weight, g (95% CI)**^**b**^
	**Rate (%)**	**OR (95% CI)**^**a**^	**Rate (%)**	**OR (95% CI)**^**a**^	**Rate (%)**	**OR (95% CI)**^**b**^	
None (n = 1356)	7.6	1.00	3.5	1.00	7.8	1.00	3008.7 (2991.6 - 3025.8)
< 1.0 g/day (n = 137)	7.3	0.98 (0.46 - 1.85)	5.8	1.69 (0.72 - 3.51)	5.8	0.75 (0.33 - 1.50)	3011.2 (2957.2 - 3065.3)
≥ 1.0 g/day (n = 72)	9.7	1.31 (0.52 - 2.84)	9.7	2.58 (1.004 - 5.80)	11.1	1.41 (0.60 - 2.94)	2952.0 (2876.6 - 3027.3)
*P* for trend		0.64		0.03		0.74	0.26

^a^Adjustment for maternal age; region of residence; number of children; family structure; maternal education; maternal employment; body mass index; maternal smoking during pregnancy; and baby’s gender.

^b^Adjustment for maternal age; region of residence; number of children; family structure; maternal education; maternal employment; body mass index; maternal smoking during pregnancy; gestational age; and baby’s gender.

In a subgroup restricted to 1427 women who had never smoked during pregnancy, the adjusted ORs for PTB for maternal alcohol consumption of less than 1.0 g and 1.0 g or more per day during pregnancy were 1.47 (95% CI: 0.55 - 3.31) and 1.73 (95% CI: 0.41 - 5.08), respectively (*P* for trend = 0.27). In a subgroup of 138 women who had smoked during pregnancy, on the other hand, the corresponding figures were 4.76 (95% CI: 0.47 - 40.13) and 15.11 (2.22 - 142.12), respectively (*P* for trend = 0.004). There was no significant interaction between maternal alcohol consumption of 1.0 g or more per day and maternal smoking during pregnancy with respect to the risk of PTB (*P* for interaction = 0.27).

## Discussion

The present study found that maternal alcohol consumption during pregnancy of 1.0 g or more per day was significantly associated with an increased risk of PTB but not LBW or SGA, and that there was no evident relationship between maternal alcohol consumption and birth weight. A prospective study of 2714 US mother-child pairs showed that light maternal drinking, defined as 0.10 oz or less of absolute alcohol per day, during month 7 was significantly positively associated with the risk of LBW and PTB but not intrauterine growth retardation, whereas mild maternal drinking, defined as > 0.10 to 0.25 oz per day, was significantly positively related to only PTB [4]. These findings are in partial agreement with our results. Moderate alcohol consumption during pregnancy was significantly associated with an increased risk of LBW and intrauterine growth retardation, but not PTB, in 1233 US women [3]. Light alcohol consumption (one to two drinks per week) during pregnancy was significantly inversely associated with the risk of LBW, PTB, or SGA in a study of 40,445 Canadian women [7]. In a case–control study in Spain, alcohol consumption of less than 6 g per day during pregnancy was significantly related to a reduced risk of LBW [8]. These findings are at variance with our results. The results of a recent meta-analysis showing no associations between light to moderate alcohol consumption during pregnancy and LBW, PTB, or SGA are not consistent with the current results regarding PTB, but are consistent with the current results regarding LBW and SGA [2].

This study has some methodological limitations that should be considered. Data on alcohol consumption were derived from the DHQ, which could only approximate consumption and was designed to assess consumption for one month prior to completion of the questionnaire. In addition, alcohol consumption assessment was carried out anywhere from the 5th to the 39th week of pregnancy. When subjects were divided into two groups according to gestation at baseline, the proportion of drinkers was 11.4% in women who had completed the baseline survey between the 5th and 17th weeks of pregnancy (n = 789) and 15.3% in women who had completed the baseline survey between the 18th and 39th weeks of pregnancy (n = 776). The adjusted OR for PTB for maternal alcohol consumption of 1.0 g or more per day during pregnancy was 2.47 (95% CI: 0.67 - 7.23, *P* for trend = 0.21) in women who had completed the baseline survey between the 5th and 17th weeks of pregnancy and 3.23 (95% CI: 0.67 - 11.59, *P* for trend = 0.03) in women who had completed the baseline survey between the 18th and 39th weeks of pregnancy. In women who had completed the baseline survey prior to the 18th week of pregnancy, the proportion of drinkers might be underestimated; in that case, the association between maternal alcohol consumption and PTB might be attenuated. Any non-differential exposure misclassification would bias the results toward the null. Moreover, information on the timing of alcohol use and of certain consumption patterns such as binge drinking was not available in the present study.

In the baseline survey, we could not estimate the participation rate because we do not have exact figures for the number of pregnant women who were provided with a set of leaflets explaining the KOMCHS, an application form, and a self-addressed and stamped return envelope by the 423 collaborating obstetric hospitals. Nevertheless, the participation rate must have been fairly low, given that the present study used data from only 970 pregnant women who lived in Fukuoka Prefecture, while, according to the government of Fukuoka Prefecture, the number of childbirths was 46,393 in 2007 and 46,695 in 2008. Furthermore, our subjects were probably not representative of Japanese women in the general population. For example, a population census conducted in 2000 in Fukuoka Prefecture found that the percentages of women aged 30 to 34 years with < 13, 13–14, ≥ 15, and an unknown number of years of education were 52.0%, 31.5%, 11.8%, and 4.8%, respectively [17]. The corresponding figures for this study were 23.1%, 33.4%, 43.5%, and 0.0%, respectively. Thus, our study subjects were more educated and probably more aware of health topics than women in the general population. Nevertheless, alcohol consumption during pregnancy in our study population was likely to be similar to that in the general population. In a study of 689 Japanese mothers whose children had undergone four-month checkups publicly provided by a municipality in 2007, the prevalence of alcohol consumption during pregnancy was 9.1% [18].

The current study did not have substantial statistical power although a significant association was detected. According to the statistical power calculation using QUANTO version 1.2 [19], our sample size gives us 48% of the power needed to detect an association between maternal alcohol consumption of 1.0 g or more per day during pregnancy and PTB.

In our population, a significant positive association was found between maternal smoking during pregnancy and PTB [20]. The positive association between maternal alcohol consumption during pregnancy and PTB was stronger in women who had smoked during pregnancy than in women who had never smoked during pregnancy; however, the interaction was not statistically significant. Although adjustment was made for several confounding factors, residual confounding effects could not be ruled out. Data on stress and family circumstances were not available in this study.

## Conclusions

A recent meta-analysis regarding maternal alcohol consumption and birth outcomes did not include epidemiological studies conducted in Asian countries [2]. To our knowledge, the present study is the first study in Japan to show that maternal alcohol consumption of 1.0 g or more per day during pregnancy was significantly positively associated with the risk of PTB but not LBW or SGA. Further epidemiological investigations are required to ascertain whether the positive association between light to moderate maternal alcohol consumption during pregnancy and the risk of PTB is replicated in other populations, especially Asian populations. Given the present results, women should abstain from alcohol as soon as possible after conception, and ideally even in the preconception period.

## Abbreviations

CI: Confidence interval; DHQ: Diet history questionnaire; KOMCHS: Kyushu Okinawa Maternal and Child Health Study; LBW: Low birth weight; OR: Odds ratio; PTB: Preterm birth; SGA: Small-for-gestational-age.

## Competing interests

All authors declare that they have no competing interests.

## Authors’ contributions

YM, KT, and MA contributed to the study concept and design and the acquisition of data. HO and SS were responsible for the estimation of dietary factors. YM was responsible for the analysis and interpretation of data and the drafting of the manuscript. All authors participated in critically revising the manuscript and approved the final version of the manuscript.

## Pre-publication history

The pre-publication history for this paper can be accessed here:

http://www.biomedcentral.com/1471-2393/14/79/prepub

## References

[B1] Henderson J Gray R Brocklehurst P Systematic review of effects of low-moderate prenatal alcohol exposure on pregnancy outcome BJOG 2007 114 243 252 10.1111/j.1471-0528.2006.01163.x 17233797

[B2] Patra J Bakker R Irving H Jaddoe VW Malini S Rehm J Dose–response relationship between alcohol consumption before and during pregnancy and the risks of low birthweight, preterm birth and small for gestational age (SGA)-a systematic review and meta-analyses BJOG 2011 118 1411 1121 10.1111/j.1471-0528.2011.03050.x 21729235 PMC3394156

[B3] Windham GC Fenster L Hopkins B Swan SH The association of moderate maternal and paternal alcohol consumption with birthweight and gestational age Epidemiology 1995 6 591 597 10.1097/00001648-199511000-00005 8589089

[B4] Lundsberg LS Bracken MB Saftlas AF Low-to-moderate gestational alcohol use and intrauterine growth retardation, low birthweight, and preterm delivery Ann Epidemiol 1997 7 498 508 10.1016/S1047-2797(97)00081-1 9349918

[B5] Aliyu MH Wilson RE Zoorob R Brown K Alio AP Clayton H Salihu HM Prenatal alcohol consumption and fetal growth restriction: potentiation effect by concomitant smoking Nicotine Tob Res 2009 11 36 43 10.1093/ntr/ntn014 19246439

[B6] Aliyu MH Lynch O Belogolovkin V Zoorob R Salihu HM Maternal alcohol use and medically indicated vs. spontaneous preterm birth outcomes: a population-based study Eur J Public Health 2010 20 582 587 10.1093/eurpub/ckq036 20375023

[B7] McDonald AD Armstrong BG Sloan M Cigarette, alcohol, and coffee consumption and prematurity Am J Public Health 1992 82 87 90 10.2105/AJPH.82.1.87 1536341 PMC1694404

[B8] Mariscal M Palma S Llorca J Pérez-Iglesias R Pardo-Crespo R Delgado-Rodríguez M Pattern of alcohol consumption during pregnancy and risk for low birth weight Ann Epidemiol 2006 16 432 438 10.1016/j.annepidem.2005.07.058 16257229

[B9] Ogawa H Tominaga S Hori K Noguchi K Kanou I Matsubara M Passive smoking by pregnant women and fetal growth J Epidemiol Community Health 1991 45 164 168 10.1136/jech.45.2.164 2072077 PMC1060736

[B10] Maruoka K Yagi M Akazawa K Kinukawa N Ueda K Nose Y Risk factors for low birthweight in Japanese infants Acta Paediatr 1998 87 304 309 10.1111/j.1651-2227.1998.tb01442.x 9560038

[B11] Nagata C Iwasa S Shiraki M Sahashi Y Shimizu H Association of maternal fat and alcohol intake with maternal and umbilical hormone levels and birth weight Cancer Sci 2007 98 869 873 10.1111/j.1349-7006.2007.00464.x 17428259 PMC11158880

[B12] Miyake Y Tanaka K Okubo H Sasaki A Arakawa M Fish and fat intake and prevalence of depressive symptoms during pregnancy in Japan: baseline data from the Kyushu Okinawa maternal and child health study J Psychiatr Res 2013 47 572 578 10.1016/j.jpsychires.2013.01.012 23391129

[B13] Sasaki S Yanagibori R Amano K Self-administered diet history questionnaire developed for health education: a relative validation of the test-version by comparison with 3-day diet record in women J Epidemiol 1998 8 203 215 10.2188/jea.8.203 9816812

[B14] Sasaki S Ushio F Amano K Morihara M Todoriki T Uehara Y Toyooka T Serum biomarker-based validation of a self-administered diet history questionnaire for Japanese subjects J Nutr Sci Vitaminol 2000 46 285 296 10.3177/jnsv.46.285 11227800

[B15] Science and Technology Agency Standard Tables of Food Composition in Japan, Fifth Revised and Enlarged Edition 2005 Tokyo: Printing Bureau of the Ministry of Finance (in Japanese)

[B16] Itabashi K Fujimura M Kusuda S Tamura M Hayashi T Takahashi T Goishi K Nimura M Takahashi Y Isobe K Iida K Uetani Y Kondo Y Shirahata S Sugiura M Takahashi N Funato M Horiuchi T Yamaguchi S Introduction of new neonatal standard anthropometric measurements Nihon Shounikagakkai Zasshi 2010 114 1271 1293 (in Japanese)

[B17] Statistics Bureau, Ministry of Public Management, Home Affairs, Posts and Telecommunications 2000 Population Census of Japan, Vol. 3-2-40, Labour Force Status of Population, Industry (Major Groups) of Employed Persons, and Education: Fukuoka-ken 2002 Tokyo: Statistics Bureau, Ministry of Public Management, Home Affairs, Posts and Telecommunications (in Japanese)

[B18] Matsumura T Taniguchi C Hamagashira N Current state of smoking and alcohol drinking among pregnant women in Kyoto City Nihon Koshu Eisei Zasshi 2009 56 655 661 (in Japanese) 19891365

[B19] Gauderman WJ Sample size requirements for matched case–control studies of gene-environment interaction Stat Med 2002 21 35 50 10.1002/sim.973 11782049

[B20] Miyake Y Tanaka K Arakawa M Active and passive maternal smoking during pregnancy and birth outcomes: the Kyushu Okinawa maternal and child health study BMC Pregnancy Childbirth 2013 13 157 10.1186/1471-2393-13-157 23919433 PMC3750375

